# The Role of Hi-Tech Devices in Assessment of Corneal Healing in Patients with Neurotrophic Keratopathy

**DOI:** 10.3390/jcm11061602

**Published:** 2022-03-14

**Authors:** Leandro Inferrera, Emanuela Aragona, Adam Wylęgała, Antonio Valastro, Gianluigi Latino, Elisa I. Postorino, Romana Gargano, Bogusława Orzechowska-Wylęgała, Edward Wylęgała, Anna M. Roszkowska

**Affiliations:** 1 Eye Clinic, Department of Medical, Surgical Sciences and Health, University of Trieste, 34100 Trieste, Italy; inferreraleandro@gmail.com; 2 Ophthalmology Clinic, Department of Biomedical Sciences, University of Messina, 98100 Messina, Italy; antonio.valastro2@gmail.com (A.V.); gianluigilatino@hotmail.it (G.L.); elisapostorino@virgilio.it (E.I.P.); aroszkowska@unime.it (A.M.R.); 3 Ophthalmology Clinic, IRCCS San Raffaele Scientific Institute, Vita-Salute San Raffaele University, 20132 Milan, Italy; aragona.emanuela@hsr.it; 4 Health Promotion and Obesity Management Unit, Pathophysiology Department, School of Medicine, Medical University of Silesia, 40-055 Katowice, Poland; 5 Department of Economics, University of Messina, 98100 Messina, Italy; romana.gargano@unime.it; 6 Clinic of Otolaryngology, Head and Neck Surgery, Department of Pediatric Surgery, Medical University of Silesia, 40-055 Katowice, Poland; edwardwylegala@interia.pl; 7 Chair and Clinical Department of Ophthalmology, Division of Medical Science in Zabrze, Medical University of Silesia, 40-055 Katowice, Poland; wylegala@gmail.com; 8 Ophthalmology Department, Faculty of Medicine and Health Sciences, Andrzej Frycz Modrzewski Krakow University, 30-705 Krakow, Poland

**Keywords:** AS-OCT, Keratograph 5M, high-tech diagnostics, neurotrophic keratopathy, rh-NGF, persistent epithelial defects, corneal ulcers, corneal healing

## Abstract

To prove the role of high-tech investigation in monitoring corneal morphological changes in patients with neurotrophic keratopathy (NK) using Keratograph 5M (K5M) and anterior segment OCT (AS-OCT), corneal healing was monitored with Keratograph 5M (Oculus, Wetzlar, Germany) and AS-OCT (DRI, Triton, Topcon, Tokyo, Japan) in 13 patients (8F and 5M), aged from 24 to 93 years (67.8 ± 19) with severe NK, who were treated with Cenegermin 0.002% (20 μg/mL) (Oxervate^®^, Dompè, Farmaceutici Spa, Milan, Italy). The surface defects were evaluated on Keratograph 5M with ImageJ software and the corneal thickness variations were measured using DRI-Triton OCT software. Instrumental procedures were performed at baseline, and after 4 and 8 weeks of the treatment, respectively. The main outcome measures were reduction of the ulcers’ area and corneal thickness recovery. The mean area of the corneal ulcers was reduced between baseline and 4 weeks examination in all patients, and at 8 weeks all ulcers were completely healed. An increase of the corneal thickness was evidenced between the baseline visit and after the 4- and 8-week follow-up, respectively. Additionally, only in collaborating subjects the In Vivo Confocal Microscopy (IVCM) was performed with HRT Rostock Cornea Module (Heidelberg Eng GmbH) to study the corneal nerves fibres. High-tech diagnostics with K5M, AS-OCT and IVCM proved useful in the assessment of corneal morphology and the healing process in patients with NK and could be extended to assess other corneal pathologies.

## 1. Introduction

Unstoppable technological advances during previous decades have introduced new diagnostic tools in ophthalmology that provide high-level information about ocular structure and function. These cutting-edge tools for cornea and ocular surface investigation permit assessment and quantification of corneal morphological changes during the healing processes and in relation to the adopted therapies.

Anterior segment optical coherence tomography (AS-OCT), based on swept-source laser technology, is an imaging technique for investigation of the anterior segment of the eye, allowing better evaluation, diagnosis, and management of different corneal diseases [1,2,3].

AS-OCT measures the entire corneal and epithelial thickness, analyzes corneal deposits and infiltrates, providing information as to their location and stromal depth, and enables the evaluation of the corneal flap following LASIK. It is also crucial in corneal lamellar surgery and in the study of ectatic pathologies, and a proven utility in the evaluation of pterygium, pinguecula, and scleromalacia [2,4,5,6,7,8]. Different studies have highlighted the role of AS-OCT in the management of corneal infections such as keratitis and peripheral ulcers [9,10,11,12].

Keratograph 5M (OCULUS Optikgeräte GmbH, Wetzlar, Germany) is a video topographer and a non-invasive, repeatable, and reproducible tool for the assessment of the ocular surface, allowing the analysis of different morpho-functional aspects. It combines corneal topography and dry eye study in a single tool and represents the gold standard for ocular surface investigation [13,14,15,16,17,18]. A series of studies has demonstrated the importance and effectiveness of Keratograph 5M (K5M) in the evaluation of ocular surface diseases, such as Dry Eye Disease (DED) and Meibomian Gland Dysfunction (MGD) [13,15,16].

In vivo corneal confocal microscopy (IVCM) represents the main tool in the clinical investigation of the cornea in health and disease. It allows visualization of all corneal layers, cells, and nerves with extreme precision and high resolution that can be compared with histological imaging.

The normal SBNP fibers can be easily detected and appear as straight, well-defined, and clearly identified reflective structures that run in parallel and exhibit different grades of branching, tortuosity, and beadings [19].

The opportunity to examine, in vivo, the corneal sub-basal nerve plexus with a fast and repeatable method, and to perform a qualitative and quantitative analysis, makes the IVCM an important diagnostic tool to investigate the nerve fiber’s status in corneal and systemic diseases [20,21].

Neurotrophic keratitis (NK) is caused by damage to the corneal innervation at any level (from the brain to the nerve endings of the sub-basal corneal nerve plexus), resulting in a reduction or abolition of corneal sensitivity, and alteration of the corneal trophism ensured by the proper innervation [22,23]. If untreated, it can compromise corneal healing, resulting in recurrent corneal erosions and, consequently, an increased risk of corneal over-infection, melting, and perforation [5].

There are many causes that can lead to neurotrophic keratitis (NK), including local or systemic diseases, surgery, trauma, infectious, and metabolic diseases; however, herpetic keratitis is the most frequently encountered cause [24,25].

In 1995, Mackie proposed the first classification of NK, with stages I, II, and III. Stage I is characterized by epithelial irregularity, such as punctate keratopathy; stage II is defined by recurrent or persistent epithelial defects (PED) without stromal involvement; and stage III is characterized by stromal involvement with ulcer, melting, and perforation [26]. Dua et al. classified the pathology into mild, involving only slight epithelial changes; moderate, with persisting epithelial defects (PED); and severe, with different grades of stromal involvement [23,24,27].

There is only one report highlighting the role of AS-OCT in NK assessment, together with confocal microscopy findings and used for classification purposes. Nevertheless, to our knowledge, there are no studies on the utility of AS-OCT and K5M in the monitoring of corneal healing processes in patients with corneal degenerations, particularly in severe NK, where the important corneal defects might occur.

The aim of this study is to analyze the morphological changes occurring during the healing of the corneal ulcers in patients with severe NK treated with rh-NGF using AS-OCT and K5M.

## 2. Materials and Methods

We analyzed instrumental data of patients with severe NK treated in the Ocular Surface diseases section of the Ophthalmology Clinic of the University Hospital of Messina. Thirteen patients (8 F and 5 M), aged from 24 to 93 years (67.8 ± 19) were considered.

The diagnosis and grade of severity of NK were made with slit-lamp biomicroscopy and Cochet-Bonnet aesthesiometer, and the ocular and/or systemic pathologies that caused NK were identified. The patients received Cenegermin drops (20 μg/mL) (Oxervate^®^, Dompè, Farmaceutici Spa, Milan, Italy) 1 drop 6 times daily for 8 weeks, accordingly to the standardized protocol (https://www.ema.europa.eu/en/documents/product-information/oxervate-epar-product-information_it.pdf). The study was conducted with respect to tenets of the Declaration of Helsinki and obtained approval from the Ethical Committee of the University Hospital of Messina.

The ocular surface and corneal morphology were examined by the same physician (LI) with Keratograph 5M (OCULUS Optikgeräte GmbH, Wetzlar, Germany) and the anterior segment module of the Swept Source OCT (AS-OCT, DRI Triton, Topcon, Japan); exams were performed at baseline and repeated after 4 weeks and after 8 weeks (end of treatment). Additionally, the IVCM was performed in cooperating patients with the Laser Scanning Confocal Microscope represented by Heidelberg retina tomograph (HRT) Rostock Cornea Module (Heidelberg Eng GmbH) aimed to detect the corneal nerve fibers.

The area of corneal defect was evaluated on K5M images through fluorescein staining with ImageJ software (imagej.nih.gov/ij/index.html) and expressed in microns squared. The corneal thickness was measured on corneal images acquired with AS-OCT and expressed in microns. The corneal thickness was calculated in the thinnest corneal point, corresponding to the ulcer bed using the ruler included in the software. The mean values of three consecutive measurements were used for analysis.

The main outcome measures were related to the healing process and comprised reduction of the area of ulcer and recovery of the corneal thickness after 4 and 8 weeks of the treatment.

Numerical data were expressed as median, first and third quartile and categorical variables as numbers.

A non-parametric approach was adopted for the data analysis using the exact significance of the *p* value at the 0.050 level. This was due to the small sample size and the results of the Kolmogorov test, which revealed that the data examined were not normally distributed.

The Friedman test was applied to compare measurements at different times in each group. Wilcoxon’s test was used for pair comparisons in each subsequent session.

The statistical analyses were performed using SPSS 26.0 for Windows statistical software (SPSS Inc., Chicago, IL, USA).

## 3. Results

The main cause of NK in examined patients was represented by herpes virus keratitis determined in 6 eyes (46.1%), followed by diabetes in 2 eyes (15.3%), post-neuroma surgery in 1 eye (7.7%), post-traumatic in 1 eye (7.7%), post-surgery in 1 eye (7.7%), related to rheumatoid arthritis in 1 eye (7.7%), and to atopic conjunctivitis in 1 eye (7.7%).

### 3.1. Area of Ulcers

This group differs in the area levels recorded in the three study visits (χ2(2) = 14.89; *p* < 0.001). At the eighth week, all corneal defects healed. In the Median (IRQ) area, levels on the first day and the fourth week were 1,694,333 (628,218 to 5,214,884), 0 (0 to 1,370,276), respectively.

Post hoc analysis showed there were not statistically significant differences between week 4 and week 8 follow-up for area levels (*p* = 0.091). However, there was a statistically significant decrease in area levels in 4 and 8 weeks in comparison to baseline (*p* = 0.012, *p* < 0.011, respectively).

**Figure 1:**
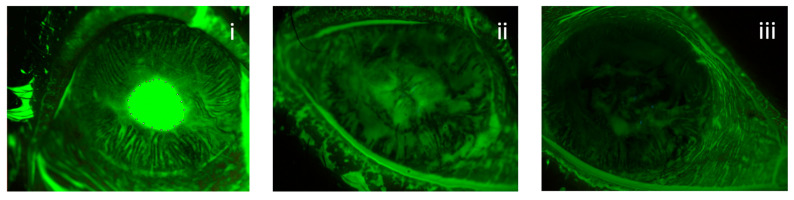
Reduction in ulcer’s area in a patient with post-herpetic NK treated with rh-NGF. The extension of the defect was calculated with ImageJ software at baseline (**i**), 4 weeks (**ii**) and 8 weeks (**iii**).

According to K5M data, after 4 weeks of the treatment, the ulcers healed completely in 69% (9 of 13) of eyes and at 8 weeks a complete recovery was registered in 100% of eyes (13 of 13) (Figure 1).

### 3.2. Corneal Thickness

**Figure 2:**
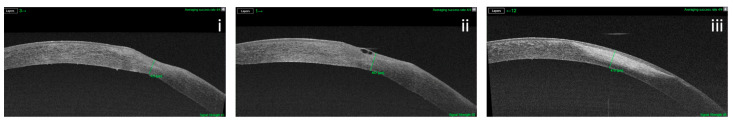
Variation of ulcer’s depth in a patient with post-herpetic NK treated with rh-NGF. The corneal thickness was calculated with OCT software at baseline: (**i**) 4 weeks, (**ii**) and 8 weeks (**iii**).

This group differs in the OCT levels recorded in the three times (χ2(2) = 14.00; *p* = 0.001). Median (IRQ) OCT levels in the three time were 333 (305 to 382), 401 (381 to 423), and 454 (432 to 586), respectively. The subsequent post hoc analysis showed statistically significant differences in all three follow-up times considered, with an increase in OCT levels (*p* = 0.015 first time vs. 4 weeks; *p* = 0.016 first time vs. 8 weeks and *p* = 0.016 4 weeks vs. 8 weeks). (Figure 2).

### 3.3. Corneal Nerves

The confocal examination was performed only in three cooperating patients at baseline and in five subjects at 8 weeks at the end of the treatment. The examination was unexecutable in other participants.

**Figure 3:**
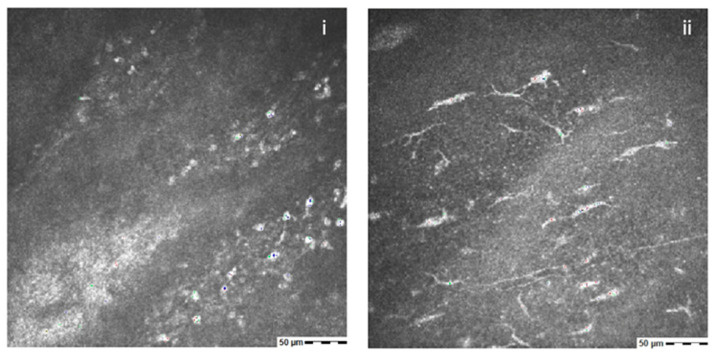
IVCM in a patient with post-herpetic NK shows no sub-basal nerve fibers at baseline (**i**). After 8 weeks of the treatment with rh-NGF, the presence of nerves can be detected (**ii**).

The total absence of nerves was observed at baseline examination, whereas after 8 weeks in four eyes the presence of sporadic fibers was detected (Figure 3).

## 4. Discussion

Several studies reported the utility of high-tech instruments in the assessment of corneal and ocular surface pathologies. However, to our knowledge, there are no reports that demonstrate the benefit of these instruments in monitoring morphological changes occurring in corneal tissue in patients with NK.

Optical coherence tomography (OCT) is a non-contact, in vivo imaging technique that uses low coherence interferometry to obtain in vivo imaging of different ocular structures [28].

As applied to cornea with an anterior segment module, the OCT imaging confirmed its crucial role in the clinical examination of the cornea and anterior segment of the eye.

Several studies demonstrated the usefulness of the AS-OCT in the clinical evaluation of a large number of corneal pathologies and in assessing the differences between normal and abnormal epithelium in the ocular surface neoplasia [29,30,31]. The undisputed role of AS-OCT in corneal assessment was reported in cases of edema, corneal infiltrates in microbial keratitis, and in peripheral ulcers [9,10,11,12,22]. AS-OCT also became a fundamental tool in the study of stromal deposits and structural changes in corneal dystrophies and degenerations and in the evaluation of the progression of keratoconus [32,33,34,35,36,37,38,39]. However, to our knowledge, the present study represents an initial report of the meaningful role of AS-OCT in the monitoring of corneal structural changes in the eyes with severe NK as the response to the therapy [40].

In the present study, we used AS-OCT to analyze the corneal thickness changes in the response to the treatment with Cenegermin in patients with severe NK. The results showed a significant increase of the corneal thickness that permitted us to consider the importance of imaging in monitoring the healing process.

K5M is a video topographer that allows analysis of tear meniscus height, lipid layer interferometry, NIBUT, bulbar redness, and/or Meibomian glands through Meibo-Scan in an automated, non-invasive, and objective manner [13]. Several studies highlighted the importance of K5M in evaluation and follow-up of patients with Dry Eye disease, but no studies reported the utility of K5M in monitoring the healing of corneal defects in patients with NK [14,15,16,17].

IVCM is the main tool in the structural investigation of the cornea. It allows visualization of all corneal layers, with cells and nerves, with a high resolution that can be compared with histological imaging. Specifically, it offers the opportunity to study the sub-basal nerve plexus fibers responsible for corneal health and trophism [19].

Neurotrophic keratitis (NK) is a progressive, degenerative condition that is characterized by diminished corneal sensitivity and inadequate corneal repair. This condition makes the cornea more vulnerable to damage and reduces the tearing reflex. As a result of inadequate healing, epithelial breakdown may result in ulceration, infection, melting, and perforation [23].

NK may be caused by any permanent modification of the corneal sensory innervation that impairs the function of the post-ganglionic fibers [41].

Numerous ocular or systemic disorders, such as diabetes mellitus or neurosurgical surgeries, may damage the V nerve, but herpes virus infection is considered as the primary cause of NK, resulting in the loss of sensory ganglion fibers and/or cells [23,41,42,43].

In our study, Herpes virus keratitis was found as the primary local cause of NK, followed by diabetes, using data consistent with those found in the literature [23].

Although the Mackie classification has been used for several years, Dua et al. proposed another adaption that is more therapeutically useful and includes severity and prognosis information. This classification divides the extent of pathology into: mild (characterized by epithelial irregularity without a true epithelial defect), moderate (characterized by epithelial defect without stromal defect), and severe, which is characterized by stromal involvement ranging from corneal ulceration to lysis and perforation, accompanied by corneal hypo-anaesthesia [23]. In 2019, Mastropasqua et al. proposed another classification of NK based on the sub-basal nerve fiber layer assessed with in vivo confocal microscopy (IVCM) and the stromal structure, evaluated with AS-OCT.

In their report, the authors highlighted the role of the high-tech instruments in the assessment of the corneal structure in NK, however, without follow up and quantification of data [44].

In the present study, we measured the extension and depth of the corneal defects, and we were able to provide a statistical assessment of the morphological changes occurring in the corneal tissue as a response to the therapy.

Different therapeutic approaches are used in relation to the stage of NK and include artificial tears, topical autologous serum, RGTA-a matrix, thyroxin beta-4, topical substance P, and insulin-like GF1 [45,46,47,48,49,50,51,52,53].

The treatment milestone was achieved when recombinant human nerve growth factor (rhNGF) was introduced and after proved efficacy in clinical trials approved by EMA and FDA.

Currently, rhNGF (Cenegermin, Dompe, Milan, Italy) represents the first-line medical treatment for moderate and severe forms of NK and acts to restore the corneal nerves and consequently, corneal trophism and epithelial healing [44,54,55,56,57].

The efficacy and safety of treatment with rh-NGF were reported in clinicals where the corneal defects extension was assessed using fluorescein staining [55,56,58]. In our study, we report the morphological corneal changes evidenced with new generation instruments such as AS-OCT and K5M during the Cenegermin administration in patients with NK ulcers. Both devices provided us with detailed information on the healing processes, improving our knowledge on corneal changes induced by therapy.

The IVCM allowed us to demonstrate the nerve’s regrowth after the treatment, confirming the effectiveness of rhNGF in promoting corneal reinnervation in NK. Similarly, Mastropasqua et al. confirmed the ability of Cenegermin to heal persistent epithelial defects and corneal ulcers in a group of patients with NK, assessing the corneal nerves with confocal investigation [59].

The importance of IVCM in monitoring the effects of corneal healing and nerves restoration after neurotization in NK was also reported by Fung et al. [60].

The presented results proved the possibility of qualitative and quantitative analysis of corneal defects, allowing the specialist to follow the changes during the treatment.

Additionally, the use of IVCM, even if limited, allowed us to prove the effect of the treatment on nerve restoration with the presence of new fibers in the acquired pictures.

However, this device cannot be used in all patients due to the frequent poor cooperation and invasiveness related to the contact approach in significantly affected eyes.

The use of IVCM in NK patients is of high importance, as it proves the restoration of corneal health, together with morphological improvement as assessed with Keratograph and AC-OCT.

In conclusion, in this study we demonstrated that the use of new technologies offers inestimable opportunities to accurately monitor corneal response to therapies in several diseases where deep stromal defects or different structural alterations might occur. These results are based on the use of high-tech in assessing corneal healing in severe NK with deep defects. We believe that this instrumental approach could be extended to the other corneal pathologies, i.e., infectious, degenerative, and dystrophic, providing qualitative and quantitative information on the corneal morphology.

## Data Availability

The data are available on request.

## References

[1] Ang M., Baskaran M., Werkmeister R.M., Chua J., Schmidl D., Aranha Dos Santos V., Garhöfer G., Mehta J.S., Schmetterer L. (2018). Anterior segment optical coherence tomography. Prog. Retin. Eye Res..

[2] Lim S.-H. (2015). Clinical Applications of Anterior Segment Optical Coherence Tomography. J. Ophthalmol..

[3] Venkateswaran N., Galor A., Wang J., Karp C.L. (2018). Optical coherence tomography for ocular surface and corneal diseases: A review. Eye Vis..

[4] Martin R., Sridhar M. (2018). Anterior segment optical coherence tomography for evaluation of cornea and ocular surface. Indian J. Ophthalmol..

[5] Czajkowski G., Kaluzny B., Laudencka A., Malukiewicz G., Kałużny J.J. (2012). Tear Meniscus Measurement by Spectral Optical Coherence Tomography. Optom. Vis. Sci..

[6] Soliman W., Mohamed T.A. (2012). Spectral domain anterior segment optical coherence tomography assessment of pterygium and pinguecula. Acta Ophthalmol..

[7] Randleman J.B., Woodward M., Lynn M.J., Stulting R.D. (2008). Risk Assessment for Ectasia after Corneal Refractive Surgery. Ophthalmology.

[8] Chow V.W., Agarwal T., Vajpayee R.B., Jhanji V. (2013). Update on diagnosis and management of Descemet’s membrane detachment. Curr. Opin. Ophthalmol..

[9] Konstantopoulos A., Kuo J., Anderson D., Hossain P. (2008). Assessment of the Use of Anterior Segment Optical Coherence Tomography in Microbial Keratitis. Am. J. Ophthalmol..

[10] Bonnet C., Debillon L., Al-Hashimi S., Hoogewoud F., Monnet D., Bourges J.-L., Brézin A. (2020). Anterior segment optical coherence tomography imaging in peripheral ulcerative keratitis, a corneal structural description. BMC Ophthalmol..

[11] Schwarz C., Dang Burgener N.P., Dosso A.A. (2008). OCT Visante observation of the progression of a perforated neurotrophic cornea ulcer treated with amniotic membrane grafts. J. Fr. Ophtalmol..

[12] Sheha H., Tighe S., Hashem O., Hayashida Y. (2019). Update on Cenegermin Eye Drops in the Treatment of Neurotrophic Keratitis. Clin. Ophthalmol..

[13] García-Marqués J.V., Martínez-Albert N., Talens-Estarelles C., García-Lázaro S., Cerviño A. (2021). Repeatability of Non-invasive Keratograph Break-Up Time measurements obtained using Oculus Keratograph 5M. Int. Ophthalmol..

[14] Best N., Drury L., Wolffsohn J.S. (2012). Clinical evaluation of the Oculus Keratograph. Contact Lens Anterior Eye.

[15] Wang M.T.M., Craig J.P. (2018). Comparative Evaluation of Clinical Methods of Tear Film Stability Assessment: A Randomized Crossover Trial. JAMA Ophthalmol..

[16] Tian L., Qu J.-H., Zhang X.-Y., Sun X.-G. (2016). Repeatability and Reproducibility of Noninvasive Keratograph 5M Measurements in Patients with Dry Eye Disease. J. Ophthalmol..

[17] García-Montero M., del Viejo L.R., Lorente-Velázquez A., Martínez-Alberquilla I., Hernández-Verdejo J.L., Madrid-Costa D. (2019). Repeatability of Noninvasive Keratograph 5M Measurements Associated With Contact Lens Wear. Eye Contact Lens.

[18] Alfaro-Juárez A., Caro-Magdaleno M., Montero-Iruzubieta J., Fernández-Palacín A., Muñoz-Morales A., Castilla-Martino M.A., Spínola-Muñoz C., de la Rua E.R. (2019). Keratograph 5M As a Useful and Objective Tool for Evaluating the Ocular Surface in Limbal Stem Cell Deficiency. Clin. Ophthalmol..

[19] Cruzat A., Qazi Y., Hamrah P. (2017). In Vivo Confocal Microscopy of Corneal Nerves in Health and Disease. Ocul. Surf..

[20] Roszkowska A.M., Wylęgała A., Gargano R., Spinella R., Inferrera L., Orzechowska-Wylęgała B., Aragona P. (2021). Impact of corneal parameters, refractive error and age on density and morphology of the subbasal nerve plexus fibers in healthy adults. Sci. Rep..

[21] Al-Aqaba M.A., Dhillon V.K., Mohammed I., Said D.G., Dua H.S. (2019). Corneal nerves in health and disease. Prog. Retin. Eye Res..

[22] Ruiz-Lozano R.E., Hernandez-Camarena J.C., Loya-Garcia D., Merayo-Lloves J., Rodriguez-Garcia A. (2021). The molecular basis of neurotrophic keratopathy: Diagnostic and therapeutic implications. A review. Ocul. Surf..

[23] Dua H.S., Said D.G., Messmer E.M., Rolando M., Benitez-Del-Castillo J.M., Hossain P.N., Shortt A.J., Geerling G., Nubile M., Figueiredo F.C. (2018). Neurotrophic keratopathy. Prog. Retin. Eye Res..

[24] Araki K., Ohashi Y., Kinoshita S., Hayashi K., Kuwayama Y., Tano Y. (1994). Epithelial wound healing in the denervated cornea. Curr. Eye Res..

[25] Bonini S., Rama P., Olzi D., Lambiase A. (2003). Neurotrophic keratitis. Eye.

[26] Mackie I., Fraunfelder F., Roy F.H., Meyer S.M. (1995). Neuroparalytic keratitis. 1246 Current Ocular Therapy.

[27] Sacchetti M., Lambiase A. (2014). Diagnosis and management of neurotrophic keratitis. Clin. Ophthalmol..

[28] Huang D., Swanson E.A., Lin C.P., Schuman J.S., Stinson W.G., Chang W., Hee M.R., Flotte T., Gregory K., Puliafito C.A. (1991). Optical coherence tomography. Science.

[29] Nanji A.A., Sayyad F.E., Galor A., Dubovy S., Karp C.L. (2015). High-Resolution Optical Coherence Tomography as an Adjunctive Tool in the Diagnosis of Corneal and Conjunctival Pathology. Ocul. Surf..

[30] Gasser T., Romano V., Seifarth C., Bechrakis N.E., Kaye S.B., Steger B. (2017). Morphometric characterisation of pterygium associated with corneal stromal scarring using high-resolution anterior segment optical coherence tomography. Br. J. Ophthalmol..

[31] Lluch S., Julio G., Pujol P., Merindano D. (2016). What biomarkers explain about pterygium OCT pattern. Graefes Arch. Clin. Exp. Ophthalmol..

[32] Maeda N. (2010). Optical Coherence Tomography for Corneal Diseases. Eye Contact Lens.

[33] Matalia H., Francis M., Gangil T., Chandapura R.S., Kurian M., Shetty R., Nelson E.J.R., Sinha Roy A. (2017). Noncontact Quantification of Topography of Anterior Corneal Surface and Bowman’s Layer With High-Speed OCT. J. Refract. Surg..

[34] Fuentes E., Sandali O., El Sanharawi M., Basli E., Hamiche T., Goemaere I., Borderie V., Bouheraoua N., Laroche L. (2015). Anatomic Predictive Factors of Acute Corneal Hydrops in Keratoconus: An Optical Coherence Tomography Study. Ophthalmology.

[35] Li Y., Chamberlain W., Tan O., Brass R., Weiss J.L., Huang D. (2016). Subclinical keratoconus detection by pattern analysis of corneal and epithelial thickness maps with optical coherence tomography. J. Cataract. Refract. Surg..

[36] Ortiz S., Pérez-Merino P., Alejandre N., Gambra E., Jimenez-Alfaro I., Marcos S. (2012). Quantitative OCT-based corneal topography in keratoconus with intracorneal ring segments. Biomed. Opt. Express.

[37] Pahuja N., Shroff R., Pahanpate P., Francis M., Veeboy L., Shetty R., Nuijts R.M.M.A., Sinha Roy A. (2017). Application of high resolution OCT to evaluate irregularity of Bowman’s layer in asymmetric keratoconus. J. Biophotonics.

[38] Sandali O., El Sanharawi M., Temstet C., Hamiche T., Galan A., Ghouali W., Goemaere I., Basli E., Borderie V., Laroche L. (2013). Fourier-domain optical coherence tomography imaging in keratoconus: A corneal structural classification. Ophthalmology.

[39] Su J.P., Li Y., Tang M., Liu L., Pechauer A.D., Huang D., Liu G. (2015). Imaging the anterior eye with dynamic-focus swept-source optical coherence tomography. J. Biomed. Opt..

[40] Siebelmann S., Scholz P., Sonnenschein S., Bachmann B., Matthaei M., Cursiefen C., Heindl L.M. (2018). Anterior segment optical coherence tomography for the diagnosis of corneal dystrophies according to the IC3D classification. Surv. Ophthalmol..

[41] Sacchetti M., Lambiase A. (2017). Neurotrophic factors and corneal nerve regeneration. Neural Regen. Res..

[42] Versura P., Giannaccare G., Pellegrini M., Sebastiani S., Campos E.C. (2018). Neurotrophic keratitis: Current challenges and future prospects. Eye Brain.

[43] Hamrah P., Cruzat A., Dastjerdi M.H., Zheng L., Shahatit B.M., Bayhan H.A., Dana R., Pavan-Langston D. (2010). Corneal Sensation and Subbasal Nerve Alterations in Patients with Herpes Simplex Keratitis: An In Vivo Confocal Microscopy Study. Ophthalmology.

[44] Mastropasqua L., Nubile M., Lanzini M., Calienno R., Dua H.S. (2019). In vivo microscopic and optical coherence tomography classification of neurotrophic keratopathy. J. Cell. Physiol..

[45] Turkoglu E., Celik E., Alagoz G. (2014). A Comparison of the Efficacy of Autologous Serum Eye Drops with Amniotic Membrane Transplantation in Neurotrophic Keratitis. Semin. Ophthalmol..

[46] Matsumoto Y., Dogru M., Goto E., Ohashi Y., Kojima T., Ishida R., Tsubota K. (2004). Autologous serum application in the treatment of neurotrophic keratopathy. Ophthalmology.

[47] Quinto G.G., Campos M.S.D.Q., Behrens A. (2008). Autologous serum for ocular surface diseases. Arq. Bras. Oftalmol..

[48] López-Plandolit S., Morales M.-C., Freire V., Etxebarría J., Durán J.A. (2010). Plasma Rich in Growth Factors as a Therapeutic Agent for Persistent Corneal Epithelial Defects. Cornea.

[49] Jeng B.H., Dupps W.J. (2009). Autologous serum 50% eyedrops in the treatment of persistent corneal epithelial defects. Cornea.

[50] Rao K., Leveque C., Pflugfelder S.C. (2010). Corneal nerve regeneration in neurotrophic keratopathy following autologous plasma therapy. Br. J. Ophthalmol..

[51] Aifa A., Gueudry J., Portmann A., Delcampe A., Muraine M. (2012). Topical Treatment with a New Matrix Therapy Agent (RGTA) for the Treatment of Corneal Neurotrophic Ulcers. Investig. Opthalmol. Vis. Sci..

[52] Guerra M., Marques S., Gil J.Q., Campos J., Ramos P., Rosa A.M., Quadrado M.J., Murta J. (2017). Neurotrophic Keratopathy: Therapeutic Approach Using a Novel Matrix Regenerating Agent. J. Ocul. Pharmacol. Ther..

[53] Dunn S.P., Heidemann D.G., Chow C.Y.C., Crockford D., Turjman N., Angel J., Allan C.B., Sosne G. (2010). Treatment of Chronic Nonhealing Neurotrophic Corneal Epithelial Defects with Thymosin Beta 4. Ann. N. Y. Acad. Sci..

[54] Lambiase A., Rama P., Bonini S., Caprioglio G., Aloe L. (1998). Topical Treatment with Nerve Growth Factor for Corneal Neurotrophic Ulcers. N. Engl. J. Med..

[55] Bonini S., Lambiase A., Rama P., Filatori I., Allegretti M., Chao W., Mantelli F., Bonini S., Lambiase A., Rama P. (2018). Phase I Trial of Recombinant Human Nerve Growth Factor for Neurotrophic Keratitis. Ophthalmology.

[56] Bonini S., Lambiase A., Rama P., Sinigaglia F., Allegretti M., Chao W., Mantelli F., REPARO Study Group (2018). Phase II Randomized, Double-Masked, Vehicle-Controlled Trial of Recombinant Human Nerve Growth Factor for Neurotrophic Keratitis. Ophthalmology.

[57] Di Zazzo A., Varacalli G., Mori T., Coassin M. (2022). Long-term restoration of corneal sensitivity in neurotrophic keratopathy after rhNGF treatment. Eur. J. Ophthalmol..

[58] Pflugfelder S.C., Massaro-Giordano M., Perez V.L., Hamrah P., Deng S.X., Espandar L., Foster C.S., Affeldt J., Seedor J.A., Afshari N.A. (2020). Topical Recombinant Human Nerve Growth Factor (Cenegermin) for Neurotrophic Keratopathy: A Multicenter Randomized Vehicle-Controlled Pivotal Trial. Ophthalmology.

[59] Mastropasqua L., Lanzini M., Dua H.S., D’Uffizi A., Di Nicola M., Calienno R., Bondì J., Said D.G., Nubile M. (2020). In Vivo Evaluation of Corneal Nerves and Epithelial Healing after Treatment with Recombinant Nerve Growth Factor for Neurotrophic Keratopathy. Am. J. Ophthalmol..

[60] Fung S.S.M., Catapano J., Elbaz U., Zuker R.M., Borschel G.H., Ali A. (2018). In Vivo Confocal Microscopy Reveals Corneal Reinnervation after Treatment of Neurotrophic Keratopathy with Corneal Neurotization. Cornea.

